# Notes and News

**Published:** 1894-11-03

**Authors:** 


### NOTES AND NEWS.

COLLECTED AND COMMUNICATED.

THE last meeting of the Dewsbury Infirmary Board revealed a lamentable want of organisation which, now that it has been brought into notice, should be remedied without fail. Some time ago, it appears, the honorary medical staff were reduced from six to four in number on the assurance of the medical men that they could better work with a smaller staff. Now it is stated by the Board that too much has been left to the house surgeon. It appears that no record of the attendance of the visiting physicians is kept, and that one of the most valued of the honorary staff left the neighbourhood without sending in his resignation, thus reducing the staff to three. The proceedings revealed a general absence of organisation, which, whether the medical staff are individually zealous or no, must act to the detriment of any institution.

THE General Purposes Committee of the Metropolitan Asylums Board are considering whether facilities should be given, and if so what facilities, to the medical superintendents of the various fever hospitals to make a trial of the supposed remedy for diphtheria, known as anti-toxin. In view of the steadily accumulating evidence of the utility of this treatment a letter has been addressed to the daily papers by Sir Joseph Lister appealing for funds to enable the British Institute of Public Health to provide a regular and trustworthy supply of the remedy. Sir Joseph Lister points out that the process by which it is procured is one of the utmost delicacy, and that the supplies which have hitherto been obtained from the Continent, while very costly, have been quite inadequate in amount and also variable in quality. The British Institute of Preventive Medicine has now, however, been able to produce a perfectly satisfactory preparation, and the results of its use have been of a most encouraging character. The Council have now determined to proceed on a much larger scale, and funds are asked for the purpose. It is pointed out that the injection is, at any rate, entirely free from danger, and that although the immunity produced may be short lived, it would be amply sufficient to protect those nursing a patient, so that there is likely to be a considerable demand for it for prophylactic as well as for therapeutic purposes.

THE Lincoln County Hospital has become the possessor of £12,000 through the will of Miss Annie Well, of Leasingham, near Sleaford.

SOME opposition appears to be aroused in Edinburgh by the prospect of the enforcement of regulations respecting the retail sale of milk. The articles which can be safely dealt in, at the same counters, with it have been the subjects of discussion, vendors naturally wishing the list to be longer than the authorities consider desirable. In London the objectionable practice of washing milk-cans on dirty pavements, and their subsequent drainage over open gutters, are dangers common in poor districts, a fact that has frequently been called attention to; certainly so-called dairies require rigid supervision in all such neighbourhoods.

MUCH disappointment was felt at Guildford by the inability of the Duke of Connaught to fulfil his intention of being present at the meeting in aid of the Royal Surrey Hospital. A large and influential gathering assembled, nevertheless, the Lord Lieutenant of the county being in the chair. Viscount Midleton passed the resolution: "That this meeting, recognizing the continually increasing usefulness of the Royal Surrey County Hospital during the last 28 years, is of opinion that its present position and the growing claims upon it urgently call for increased support, and pledges itself to do its utmost to raise the sum of £3,000 to enable the existing accommodation to be fully utilized and to meet other exceptional needs." Sir R. Webster seconded the resolution, which was carried unanimously. Another resolution, putting forth the urgent necessity for further annual subscriptions, was proposed by Lord Onslow, seconded by Lord Knutsford, and carried. Colonel Dolphin (treasurer) announced that the subscriptions received towards the special fund amounted to between £1,400 and £1,500.

THE *Westminster Gazette* draws attention to a dilemma of the near future, for such surely will be the position in which extreme anti-vivisectionists will find themselves if all we have so far heard about the serum treatment of diphtheria should turn out to be true. It will not in this case be a question of vaccinating or not vaccinating against a disease which may, and probably will, never actually arrive, but rather of saving or not saving a child suffering at the very moment from a mortal malady. Nor will it be possible to take advantage of the subterfuge that it is only knowledge which is already open to everyone that is to be made use of. The serum which is to be used as a remedy is the actual substance derived from the bleeding of the animal, and is only brought to a state of efficacy by dint of repeated operations, as the punctures for inoculation are called by the anti-vivisectionists. For an anti-vivisectionist parent who believes the statements which have been made in regard to the effective nature of the serum treatment, it will indeed be a ghastly thing to stand by and see a child suffer, and allow it to die deprived of its one chance of recovery, just because animals had had to be experimented on (or vivisected!) in order that the remedy might be produced; the horse (the victim!) meanwhile standing happily, munching hay in his stall, sleek, fat, and well groomed, instead of shivering on a cab rank or dragging out his days in weary labour on a slippery tram track. Truly experiments on animals have a good side if at the same time they can save the horse such misery and snatch the child from such a death.

